# Research on Discrete Dynamic Modeling of Learner Behavior Analysis in English Teaching

**DOI:** 10.1155/2022/1914996

**Published:** 2022-06-09

**Authors:** Junru Fu, Lingmei Cao

**Affiliations:** Department of Foreign Languages, Shanghai University of Finance and Economics Zhejiang College, Jinhua, Zhejiang 321000, China

## Abstract

The current English teaching mode focuses on the traditional offline teaching and online teaching. In order to solve the problems that some students are inefficient and cannot teach students according to their aptitude in the teaching process, this paper uses the big data analysis strategy based on a neural network algorithm. This paper studies the discrete dynamic modeling method of learner behavior analysis in English teaching. Firstly, it summarizes the current situation of English teaching and the research status of the hybrid application of discrete dynamic modeling technology. Secondly, combined with English teaching content and teaching objectives, through the analysis of various data of students' learning behavior, this paper evaluates students' English teaching quality from five aspects that affect the students' English teaching quality and puts forward a personalized English teaching quality evaluation model based on discrete dynamic modeling technology and learners' behavior analysis. Finally, through the practical teaching application in a university, the feasibility of the discrete dynamic English teaching model is verified. The results show that compared with the current innovative English teaching methods based on a dynamic iterative decision algorithm, the personalized discrete dynamic English teaching model based on learner behavior analysis significantly improves the quality of English teaching and students' academic performance.

## 1. Introduction

The mainstream English teaching still focuses on the traditional offline face-to-face teaching, supplemented by online network teaching, so the teaching mode is relatively single [1]. Since the teaching model innovation policies are promoted in different regions at this stage, the vigorous development of a variety of data analysis technologies has also triggered the innovative application of English Classroom Teaching [2]. Manufacturing computer-integrated manufacturing system CIMS, system scheduling, communication network system, database management system, military C3I system, and other systems are typical discrete event systems [3]. Therefore, customization and real-time have become important features of the College English classroom teaching system in China [4]. There are two kinds of modeling methods for this kind: informal modeling and formal modeling. Informal modeling technology refers to modeling the system with the help of computer technology through graphical representation that people can easily accept and understand, and then converting it into computer language, and analyzing the system through programs [5]. Based on English teaching and the actual situation of learner behavior analysis, this paper studies the modeling and flexible recruitment strategy of the high-tech knowledge-based discrete dynamic system. English teaching behavior is deeply influenced by teaching values, which are usually formed under the guidance of learning theory [6]. In this context, this paper studies the discrete dynamic modeling technology based on a neural network algorithm and the innovative English teaching method of learner behavior analysis.

Compared with the current innovative English teaching methods with dynamic iterative decision-making algorithms as the mainstream, the innovation of this paper is to improve the efficiency of English teaching by combining discrete dynamic modeling technology and neural network algorithm. On this basis, it can make full use of each student's learning behavior, learning data, and test scores in the English classroom through real-time dynamic tracking. It can realize customized analysis, quantitatively describe the similarity and coincidence degree of each learning behavior and teaching strategy with behavior analysis factors, complete the influence ranking of English classroom teaching indicators with quantitative indicators, and effectively conduct multivariate analysis.

Aiming at the problems of low teaching efficiency and poor intelligence in English teaching, this paper studies the construction of a discrete dynamic modeling method for learner behavior analysis in English teaching.  Chapter 1 briefly summarizes the background, innovation, and chapter arrangement of this study.  Chapter 2 introduces the current research status of English teaching models and influencing factors at home and abroad.  Chapter 3 analyzes the teaching classroom model. Based on Gaussian random function and Laplace feature recognition, a classroom teaching model is constructed. At the same time, the three-dimensional target matching analysis is carried out according to the students' classroom performance, and the quantitative characteristics are constructed.  Chapter 4 sets up a correlation experiment to verify the relevant indicators of the English classroom teaching model constructed in this paper, analyzes the experimental results, and draws a conclusion.

## 2. Related Work

China has developed slowly in the mode of innovation and quality evaluation of English teaching, while some foreign developed countries have good basic and phased innovative achievements in the field of English Teaching [7]. It is very necessary and important to evaluate teachers' teaching behavior through the study of English learning theory and find deep theoretical roots for teaching practice, so as to carry out English teaching activities more scientifically in the future [8]. In addition, it is more suitable to analyze the learners' motivation and behavior of their learners [9, 10].

Based on this model, the strategy of a discrete dynamic system for learners' behavior analysis in English teaching is put forward, and the sufficient conditions for the asymptotic stability of the closed-loop system for learners' behavior analysis are analyzed. The designed strategy can ensure that each state of the actual discrete dynamic system tracks the desired state, and the simulation results verify the effectiveness of the discrete dynamic modeling strategy proposed in this paper [11]. The establishment of a reasonable discrete dynamic model and computer simulation has always been an indispensable research method, which has played a great role in reducing losses, saving funds, shortening the cycle, reducing costs, and improving product quality and service quality [12, 13]. With the help of the description and solution methods of learners' behavior analysis problems provided by English teaching theory, the research on discrete dynamic modeling, analysis, control, and optimization of this kind of system has reached a quite mature and perfect state, at least in relatively simple branches such as linear time-invariant systems, and its effectiveness has been shown in practical applications [14]. According to the idea of neuron node association in neural network algorithm, researcher Zou et al. proposed a distributed parsing model for English teaching based on mixed contexts. The experimental results show that it has a strong speech discrimination ability [15]. Pejpichestakul et al. proposed that the Petri net modeling of discrete dynamic systems should be guided by scientific modeling methods and supported by powerful computer software. Deloitte & ToucheBakkenist of the Netherlands and Eindhoven University of Technology jointly developed ExSpect. The concept of domain model base and the design characteristics of supporting system analysis methods of this language make it a powerful modeling and simulation tool for complex discrete event dynamic systems [16]. Cao et al. proposal is more significant for basic systems such as large-scale computer and communication networks and airport traffic management systems. Therefore, the research on the modeling of discrete event dynamic systems is an urgent need for the current social production to achieve high efficiency and low fault operation [17]. Cao et al. proposed that when designing a feedback controller for modeling discrete dynamic systems, the time-varying perturbation of controller parameters may lead to the performance degradation of closed-loop systems, and even the stability will be destroyed. Therefore, the concept of elastic control was put forward. Elastic control in discrete modeling means that the control behavior can adapt to the changes in the internal conditions and external environment of the organization, and has a certain flexibility and anti-interference [18]. Deng et al. proposed that Petri net can describe the structure of a discrete dynamic modeling system well, and express the relationships of parallelism, synchronization, conflict, and sequence in the system, and the discrete dynamic combination model represented by graphics has the advantages of intuition, easy understanding, and easy use, and has its unique advantages in describing and analyzing concurrency phenomena [19]. Do et al. proposed that the use of the Petri net discrete dynamic model largely depends on the support of sufficient computer tools. To create, store, change, and analyze such discrete dynamic models with computers, it is necessary to choose a computer-friendly representation, which is called language [20]. Zheng et al. proposed to analyze the demand of enterprises for human resources through discrete dynamic modeling and gave a human resource planning scheme conducive to the development of enterprises. Note that most of these methods are based on discrete dynamic, static, and deterministic perspectives [21]. Santini et al. proposed to study and establish the m-sequence discrete dynamic test signal model according to the random characteristics of typical dynamic load, construct the electric energy measurement algorithm of the dynamic test signal, analyze and give the dynamic signal electric energy measurement error caused by the quantization error of IEC61850 protocol, and provide the basis for testing and evaluating the dynamic error characteristics of all digital electric energy meter [22].

Dynamic iterative decision-making algorithms are mainly differentiated teaching methods, and rarely make effective analyses by combining learners' behavior habits [23]. It does not have a wide application, and rarely has a good construction of differentiated analysis model and discrete dynamic evaluation model [24]. Therefore, it is of great significance to study English teaching based on learner behavior analysis.

## 3. Methodology

### 3.1. Research Status of Discrete Dynamic Modeling of English Learners' Behavior Analysis

In this paper, the discrete dynamic modeling is studied under the analysis of English teaching learners' behavior. Teachers' intuitive judgments in the classroom of English teaching learners' behavior analysis are sometimes wrong [25]. Because those students who have introverted and retreating problem behaviors in English classes are often obedient, their behaviors are easily overlooked, and teachers' explanations and solutions to the discovered problem behaviors are not necessarily correct. The discrete dynamic modeling of the whole system under the analysis of English learners' behavior is a kind of discrete dynamic system, which is characterized by the continuous change of the system state caused by the interaction of discrete according to certain operating rules. In this sense, DEDS has the following two basic characteristics [26]: first, DEDS is driven by discrete events, which is the performance of its system attributes; secondly, the running rules of DEDS are all artificial rules, which are the performance of its artificial attributes. Besides, in the process of discrete dynamic modeling of behavior research in English teaching, it is sometimes difficult to find out the real cause of problematic behavior by the commonly used methods such as questionnaire survey and interview, because students' answers are often unwilling or unable to reveal their true inner situation because of face, anxiety, or other psychological factors. For these common problem behaviors of discrete dynamic modeling, teachers should take various countermeasures according to their judgments, and test whether these countermeasures are effective in classroom practice, so as to further improve their strategies and methods.

### 3.2. Modeling of Learner Behavior Analysis in English Teaching

**Figure 1:**
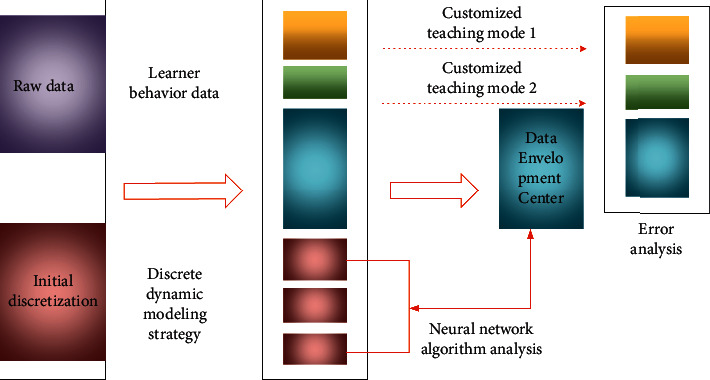
Discrete dynamic modeling method in the data analysis process of customized English teaching model.

The teaching behavior in line with the theory of “the essence of learning is trial and error” involved in sanddike's connection theory in the learning view of behavior school runs throughout the whole English teaching activities, especially in the oral training of students. English teachers often have to face the mistakes of students in language learning. This makes students inevitably make mistakes in practice. The usual practice is that no matter what mistakes students make in their expression, they will not interrupt directly, but write down the questions first. When summing up, all students are required to analyze mistakes together and constantly encourage students to speak out first, which is the first step to success. For those students who are unwilling and unable to participate in classroom activities, teachers should create more opportunities for them to express themselves, but this opportunity is selective, that is, the tasks they are competent for. Modeling is actually an iterative process, which cannot be achieved overnight. The following figure describes the important process of modeling. There are several steps in the figure, which reflect that modeling is a spiral process, and its importance is obvious. Therefore, special attention should be paid to the implementation of these steps. That is, the layer-by-layer decomposition of the system and the use of reusable components. The data analysis process is shown in Figure 1.

**Figure 2:**
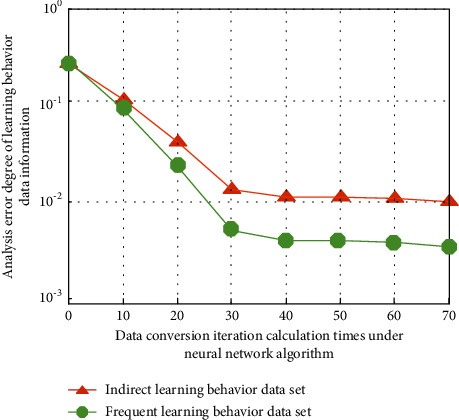
Simulation analysis results of different types of learning behavior data.

Firstly, through image sensing equipment, the current English learning behavior of different types of professional students can be recorded online. It is used in computer language processing and converts it to multi-channel binary numbers. Then the teaching system is used to store students' learning data information in a multidimensional way. Vectorization analysis and feature extraction can be carried out for the first time. In this process, simulation analysis can be carried out on the data collected by different types of learners' behaviors. The results are shown in Figure 2.

**Figure 3:**
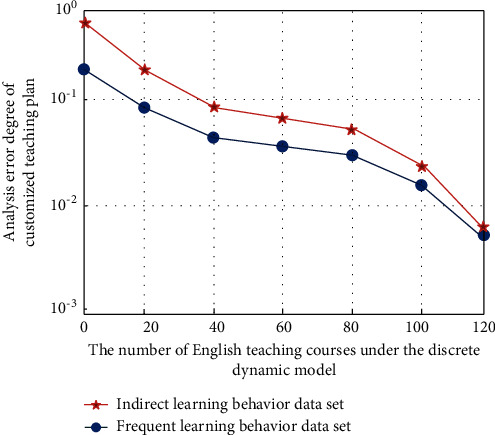
Analysis error degree of customized teaching plan based on neural network algorithm discretization dynamic modeling technology.

As can be seen from Figure 2, with the increase in the number of iterative operations in the process of converting learning behavior information into data vector information dimension, the change law of the analysis error degree of learning behavior data information is also obvious. Different learning behavior analysis methods usually separate the learning process into independent behavior operation units. It is not conducive to grasping the process and analysis of online learning activities from the overall analysis. The time series and relationship between different learning behaviors aim to reveal the learning behavior characteristics of individuals and learning groups. Identify the behavioral differences of different learning groups, diagnose key learning events in learning activities, and predict learners' learning performance. Taking English teaching learners' behavior as the research object, the purpose of this group's English learning is more important to meet the needs of work and life, and their instrumental motivation is strong. However, the self-influence factors of English learners who are under the pressure of family and friends are relatively weak. Different from basic education English learners who are forced to study English full-time under the pressure of entering a higher school, the nature of this group's part-time study of English determines that the variables of second language learning experience related to the learning environment are less influential, but the variables of English learning attitude are relatively more important. According to its characteristics, it is divided into different English teaching center models. The simulation analysis results of customized teaching optimization are shown in Figure 3.

**Figure 4:**
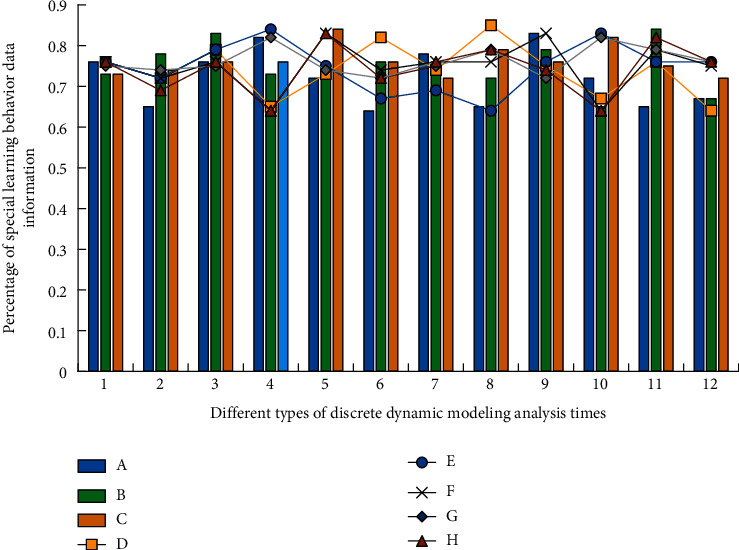
The regular relationship between different types of learner behavior data sets and special data information under the discrete dynamic model.

As can be seen from Figure 3, under the discrete dynamic modeling technology based on a neural network algorithm, with the increase in English teaching courses, the optimal customized teaching scheme has been optimized and improved to varying degrees, and shows a trend of gradual decline and then gradually stable. The improvement of students' learning enthusiasm will bring learning interest and satisfaction of success, and this cognitive internal motivation is the most important and stable motivation in classroom learning. Years of education and teaching practice have proved that students' learning enthusiasm has an obvious impact on the teaching effect. Cultivating students' learning enthusiasm is the premise and guarantee to improve teaching efficiency. The most fundamental driving force of learning comes from the enthusiasm for active learning. Therefore, due to the particularity of English learning, teachers' words and behaviors in English classes must be encouraged, and students' language output should not be criticized and attacked easily. They should adopt a tolerant attitude towards mistakes, and those students who have problematic behaviors should not be criticized and punished blindly. They must be more concerned about them and find out the causes of their problematic behaviors! Then give them continuous help and guidance. In this process, the statistical results of 8 learning behavior types (A-H) under 12 simulation analyses are shown in Figure 4.

It can be seen from the changes of statistical data in Figure 4 that with more classification of learner behavior data information (A-H), the higher the proportion of special data information in the learner's behavior data information (A-H), the greater its role, and the internal differentiation is more obvious. Although the law trend is similar, there are also obvious differences, because of the process of calculation and recognition with high similarities. The process of completing the four stages of data association in turn is defined as “frame,” that is, from the beginning of measurement sampling to the end of the physical operation and the beginning of new measurement sampling. At this stage, let *x*_*i*_=(*x*_*i*1_, *x*_*i*2_,…, *x*_*ip*_) and *x*_*j*_=(*x*_*j*1_, *x*_*j*2_,…, *x*_*jp*_) be the observed values of English learning behavior of different students, then the similarity measure function *R*(*x*) and correlation function *Q*(*x*) between them can be characterized as

(1) $$\begin{array}{c} R\left(x\right)=\frac{\left[\sum_{k=1}^{p}{\left(x_{ik}-{\bar{x}}_{i}\right)}^{2}\right]+\left[\sum_{k=1}^{p}{\left(x_{jk}-{\bar{x}}_{j}\right)}^{2}\right]}{2},\\ Q\left(x\right)=\sum_{k=1}^{p}\left(x_{ik}-{\bar{x}}_{i}\right)\left(x_{jk}-{\bar{x}}_{j}\right). \end{array}$$

The corresponding dispersion function and *W*(*x*) correlation function *T*(*x*) are

(2) $$\begin{array}{c} W\left(x\right)=\frac{\sum_{k=1}^{n}x_{ki}^{2}+\sum_{k=1}^{n}x_{kj}^{2}}{2},\\ T\left(x\right)=\sum_{k=1}^{n}\frac{x_{ki}+x_{kj}}{2}. \end{array}$$

The corresponding Fourier definite function *P*(*x*) and European iterative function *Y*(*x*) are

(3) $$\begin{array}{c} Y\left(x\right)=\sqrt{\frac{1+\left(\sum_{k=1}^{n}x_{ki}^{2}+\sum_{k=1}^{n}x_{kj}^{2}\right)}{n}},\\ P\left(x\right)=\frac{1+\left(\sum_{k=1}^{n}x_{ki}+x_{kj}/2\right)}{n}. \end{array}$$

The corresponding square sum difference *s*^2^ is

(4) $$\begin{array}{c} s_{\delta }^{1}=\frac{1}{n}{\sum_{k=1}^{n}\left[\sum_{k=1}^{n}x_{ki}^{2}+\sum_{k=1}^{n}x_{kj}^{2}\right]}^{2},\\ s_{\delta }^{2}=\frac{1}{n}{\sum_{k=1}^{n}\left[\sum_{k=1}^{n}\frac{x_{ki}+x_{kj}}{2}\right]}^{2}. \end{array}$$

### 3.3. Optimization Process of Discrete Dynamic Model Based on Neural Network Algorithm in English Teaching

**Figure 5:**
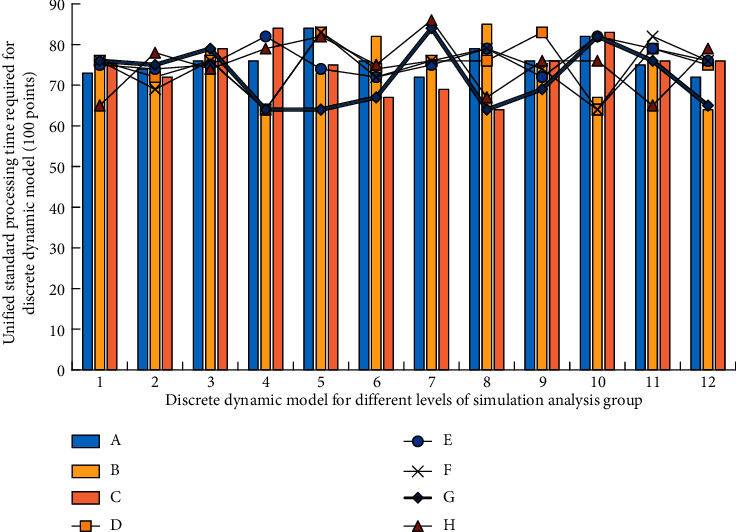
Processing time required for discrete dynamic models under different data analysis and processing groups.

In order to support the application of modeling in different fields, the concept of learner behavior analysis in English teaching is proposed. To a certain extent, this language library is similar to the professional language. The domain model library contains reusable components such as predefined system definition, mathematical function definition, and typical type definition. The use of these reusable components produces a high-level specification description of a discrete dynamic modeling system, that is, the specification and complexity of learner behavior analysis in English teaching description are reduced. Reusability is also a way to improve the efficiency of the modeling process. It can describe a discrete dynamic modeling system in less time. A discrete dynamic modeling system, which is different from a continuous dynamic system, has a large number of practical problems in the real world. The solution to such events is far from the maturity of the theory of a continuous dynamic system, which requires the further development of relevant theories. Another way is to modify the existing components in the discrete dynamic modeling system. In order to modify the existing components, users need to know the internal behavior of the components. The process of a discrete dynamic modeling system is complex and cumbersome, and sometimes it has to go through many steps of derivation. For the data of 8 learning behavior types (A-H), after 12 groups of simulation analysis are carried out on the discrete dynamic model, the data analysis and processing process are shown in Figure 5.

As can be seen from Figure 5, with the increase in data analysis and processing groups, it can be found that within a certain range (1–12), the longer the unified standard processing time is, and it is relatively stable.

(5) $$\begin{array}{c} M\left(x\right)=\frac{9x^{7}+9x^{5}+5x^{3}+3x^{2}+2}{7x^{7}+3x^{5}+5x^{3}+1},\\ L\left(x\right)=\frac{\sum_{k=1}^{n}\left(x_{ik}{\bar{x}}_{i}/i+x_{jk}{\bar{x}}_{j}/j\right)}{{\bar{x}}_{i}+{\bar{x}}_{j}},\\ Z\left(x\right)=\sqrt{\frac{\sum_{k=1}^{n}\left(x_{ik}-{\bar{x}}_{i}\right)\left(x_{jk}-{\bar{x}}_{j}\right)}{i+j}},\\ B\left(x\right)=\frac{\sqrt{\sum_{k=1}^{n}\left(x_{ik}{\bar{x}}_{i}+x_{jk}{\bar{x}}_{j}\right)}}{i+j}. \end{array}$$

The information threshold *R* corresponding to the three functions is

(6) $$\begin{array}{c} R_{L}=\frac{\sum_{k=1}^{n}\left(x_{ik}{\bar{x}}_{i}/i+x_{jk}{\bar{x}}_{j}/j\right)}{\sqrt{i^{2}+j^{2}}},\\ R_{Z}=\frac{\sum_{k=1}^{n}\left(\left(x_{ik}-{\bar{x}}_{i}\right)\left(x_{jk}-{\bar{x}}_{j}\right)\right)}{\sqrt{i^{2}+j^{2}}},\\ R_{B}=\frac{\sqrt{i^{2}+j^{2}}}{\sum_{k=1}^{n}\left(x_{ik}{\bar{x}}_{i}+x_{jk}{\bar{x}}_{j}/i+j\right)}. \end{array}$$

**Figure 6:**
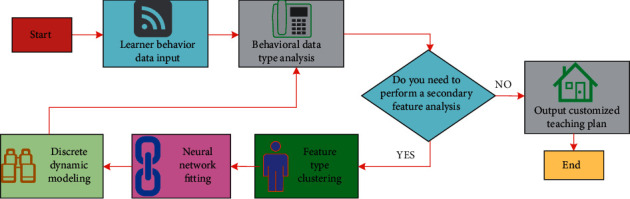
The process of analyzing and processing learner behavior data based on the discrete dynamic model of neural network algorithm.

Based on the informal description of learner behavior analysis in English teaching, we distinguish the discrete dynamic modeling system entities involved, such as suppliers, customers, and transporters of the logistics system. We decompose the logistics system into subsystems until each subsystem is a basic material system, a basic information system, a control system, or a component. It has been widely used in many fields because of its strong ability for function distribution and function description. It is a powerful tool for modeling and performance analysis of asynchronous, discrete, and concurrent event dynamic systems. The simulation analysis is carried out, and the data processing process is shown in Figure 6.

## 4. Result Analysis and Discussion

### 4.1. Experimental Process of Discrete Dynamic Model for Learner Behavior Analysis in English Teaching

**Figure 7:**
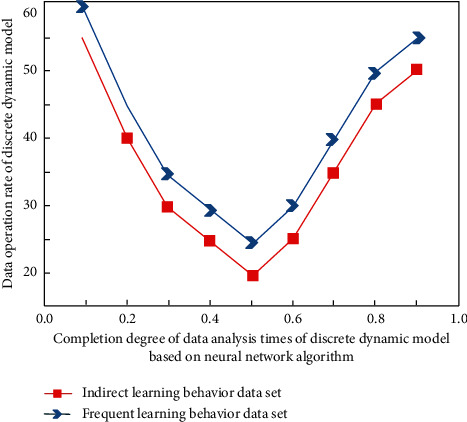
Experimental simulation analysis results.

**Figure 8:**
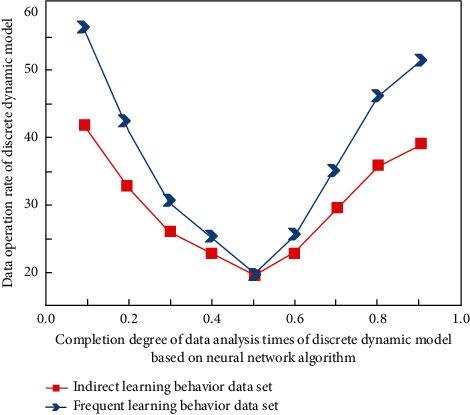
Preliminary experimental analysis results.

In order to verify the practicability of the English teaching model constructed in this study, this study carries out practical teaching experiments combined with the actual English curriculum content, teaching process, and students' learning behavior data characteristics in Colleges and universities, and makes quantitative analysis according to students' classroom performance behavior. These external and controllable factors are attributed to the very few external and controllable factors. It shows that students' attribution tendency to their success in learning English has high stability. In foreign language learning, they generally work hard and can control their learning behavior. They believe that they can accept academic challenges. Even if they have unsuccessful experiences, they will not attribute their failure to their ability. This positive attribution will encourage students to actively summarize the experience, stimulate motivation, maintain motivation level, and help them succeed in subsequent learning activities. The simulation analysis results before the experiment are shown in Figure 7, and the results in the experiment are shown in Figure 8.

It can be seen from Figures 7 and 8 that in the discrete dynamic model based on the neural network algorithm, different types of learner behavior data have different characteristics. When determining the experimental data, with the improvement of the completion degree of data analysis times, the corresponding operation and analysis speed shows a changing trend of first decreasing and then increasing, whether in the process of simulation or experiment.

The data analysis times and completion degree of discrete dynamic model based on neural network algorithm increase, and the data operation rate of discrete dynamic model decreases and then increases. These are external controllable factors, which are caused by a very small number of external controllable factors.

Great changes have taken place in its internal relevance, and its changing laws are also different. The difference between the experimental process and the simulation process lies in the difference in minimum value. This is because different simulation data sets have different comprehensive solutions and correlation analyses.

### 4.2. Experimental Results and Analysis

**Figure 9:**
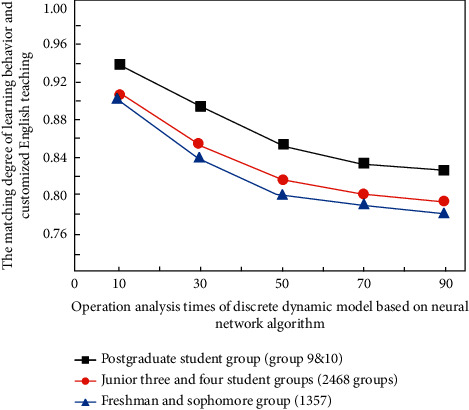
The matching degree of the corresponding learning behavior of the discrete dynamic model with the customized English teaching under different operation analysis times.

This experimental study adopts different questionnaire contents. The questionnaire includes the following aspects: English learning habits, oral practice time, English reading habits, classroom student participation, types of homework completion, and English listening status. In the overall process of the experiment, 88.3% of the people have obvious effects, of which 62% are girls and 26% are boys. The error analysis results of the experimental results are shown in Figure 9 (1–10 represents ten groups of students who use discrete dynamic modeling to analyze English teaching, including 1357 groups for freshmen and sophomores, and 2468 groups for juniors and seniors.

It can be seen from Figure 9 that in the discrete dynamic model based on a neural network algorithm, after analyzing the learning behavior of freshmen, sophomores, seniors, and postgraduates (master and doctoral) of the University, the overall teaching quality is relatively good in the process of customized English classroom teaching. Among the research variables, the average change rate of English learning attitude is the highest. Internalized instrumental motivation and ideal second language self-motivation do not produce strong learning motivation among adult English learners in open education, which shows that “the existence mode and degree of ideal second language self in various educational and learning situations are inconsistent, and there are great differences in the impact on learning behavior and learning feeling.”

## 5. Conclusion

This paper analyzes learners' behavior in English teaching. In the discrete dynamic model of students' English classroom teaching, students' behavior is constantly changing, and the causes of problem behavior are also different. Based on the analysis of English learners' behavior, a discrete dynamic model suitable for a general hybrid simulation system is established. Firstly, this paper analyzes and expounds on the current research status of English teaching. The application of dynamic modeling technology is analyzed. Secondly, combined with the English teaching content and teaching objectives, through various data analyses of students' learning behavior, this paper evaluates students' English teaching quality from five aspects that affect students' English teaching quality and puts forward a customized English teaching quality evaluation model based on discrete dynamic modeling technology and learners' behavior analysis. Finally, through the practical teaching application in a university, the feasibility of the discrete dynamic English teaching model is verified. The results show that compared with the current innovative English teaching methods dominated by dynamic iterative decision-making algorithms, the customized discrete dynamic English teaching model based on learner behavior analysis has significantly improved the quality of English teaching and the improvement of students' performance. In English teaching, they should also learn and understand the theories and research results of educational psychology, which can help teachers understand “problem-finding and problem-solving behaviors” from different aspects, fundamentally improve students' interest and enthusiasm, and truly improve the efficiency and quality of behavior analysis of English teaching learners. However, this paper does not consider the personalized teaching model according to the actual situation of different students. Therefore, the model in this paper may deviate from the actual simulation. This requires further discussion in the future.

## Data Availability

The data used to support the findings of this study are included in the article.
